# Quality Attributes and Storage of Tomato Fruits as Affected by an Eco-Friendly, Essential Oil-Based Product

**DOI:** 10.3390/plants10061125

**Published:** 2021-06-01

**Authors:** Panayiota Xylia, Irene Ioannou, Antonios Chrysargyris, Menelaos C. Stavrinides, Nikolaos Tzortzakis

**Affiliations:** Department of Agricultural Sciences, Biotechnology and Food Science, Cyprus University of Technology, 3036 Limassol, Cyprus; pa.xylia@edu.cut.ac.cy (P.X.); i.ioannou1996@gmail.com (I.I.); a.chrysargyris@cut.ac.cy (A.C.); m.stavrinides@cut.ac.cy (M.C.S.)

**Keywords:** tomato, eco-friendly product, essential oils, quality preservation, antioxidants, damage index

## Abstract

The preservation of fresh produce quality is a major aim in the food industry since consumers demand safe and of high nutritional value products. In recent decades there has been a turn towards the use of eco-friendly, natural products (i.e., essential oils-EOs) in an attempt to reduce chemical-based sanitizing agents (i.e., chlorine and chlorine-based agents). The aim of this study was to evaluate the efficacy of an eco-friendly product (EP—based on rosemary and eucalyptus essential oils) and two different application methods (vapor and dipping) on the quality attributes of tomato fruits throughout storage at 11 °C and 90% relative humidity for 14 days. The results indicated that overall, the EP was able to maintain the quality of tomato fruits. Dipping application was found to affect less the quality attributes of tomato, such as titratable acidity, ripening index and antioxidant activity compared to the vapor application method. Vapor application of 0.4% EP increased fruit’s antioxidant activity, whereas tomatoes dipped in EP solution presented decreased damage index (hydrogen peroxide and lipid peroxidation levels), activating enzymes antioxidant capacity (catalases and peroxidases). Moreover, higher EP concentration (up to 0.8%) resulted in a less acceptable product compared to lower concentration (0.4%). Overall, the results from the present study suggest that the investigated EP can be used for the preservation of fresh produce instead of the current commercial sanitizing agent (chlorine); however, the method of application and conditions of application must be further assessed for every commodity tested.

## 1. Introduction

Fresh produce is considered to be an important source of vitamins (i.e., A, C, niacin, riboflavin, thiamine), minerals (i.e., potassium, calcium, iron) and dietary fibers. Increase consumption of vegetables has been associated with a healthy lifestyle, reducing the risk of vitamin and mineral deficiencies, cancer and other chronic diseases [1]. These benefits derive from the previously mentioned phytonutrients that possess antioxidant, anti-inflammatory, and anti-cancer properties, among others [2].

Vegetables are perishable products and their quality might be affected by various environmental factors throughout the food supply chain [2]. The factors that can influence the quality and storability of vegetables include pre-harvest (i.e., growing temperature and light conditions, irrigation, maturity, pest management, harvesting, cultivation practice) and postharvest (i.e., poor handling, processing, storage temperature, marketing, pathogens) parameters [3,4,5,6]. During postharvest handling (including processing, storage, transport and retailing) and under unfavorable conditions (i.e., high temperature, low relative humidity, improper hygiene), vegetables’ quality gradually deteriorates resulting in great losses for the food industry [1,7,8].

During storage, fresh produce might exhibit water loss (wilting), degradation of pigments (discoloration, i.e., loss of chlorophylls, carotenoids), and increased susceptibility to diseases and all these result in a less acceptable product by the consumer [2,3]. The main factors that affect vegetables’ quality include exposure to undesirable temperature, relative humidity and light [2]. It has been shown that storage at low temperatures and high humidity suppresses the respiration rate of fresh produce, extending their shelf life [9]. Furthermore, the use of sanitizing agents including chlorine and chlorine-based means for fresh produce decontamination, might not be able to sufficiently reduce the microbial load, while at the same time, these products have been associated with the production of harmful, carcinogenic compounds [9,10].

Nowadays there is a turn towards the investigation of natural products in an attempt to reduce the use of chemical sanitizing agents in the food industry and meet consumers’ demands for fresh, high nutritional and safe fresh produce [11,12]. Chlorine, the most commonly used sanitizing agent, has been linked with the formation of carcinogenic compounds that can adversely affect human health [10] and its application is of concern. Among the natural products investigated, the essential oils (EOs) from medicinal and aromatic plants gained more attention by researchers due to their antioxidant, anti-inflammatory, antifungal and antibacterial activities, among others [4,11,13,14,15,16,17,18,19]. Various EOs have been used in the food industry (as food preservatives) in a variety of foods including meat and meat products, fruits and vegetables, minimally processed products and dairy products [8,19,20,21].

The application of EOs for the postharvest preservation of fresh produce and the utilization of their properties have been previously reported and the results are promising since they are able to preserve/improve product quality and ensure its safety for consumption [4,8,22]. The use of EOs alone or in combination with other compounds, i.e., chitosan, on fresh and/or minimally processed vegetables (including tomato and cucumber) has been previously studied and the results are encouraging [4,17,23,24,25]. For instance, EOs from eucalyptus lemon, helichrysum, sage, nutmeg, cinnamon and clove inhibited the growth of *Escherichia coli* in cucumber fruit, preserving fruit’s quality and flavor [18]. The use of dittany EO in eggplant fruits decreased gray mold (*Botrytis cinerea*) development and at the same time did not negatively affect fruit’s quality attributes [15]. The use of natural products (including sage EO) in vapor phase resulted in suppressed gray mold growth when inoculated on pepper fruits, while sage EO incorporated in *Aloe vera* gel improved (via dipping application) tomato fruit quality attributes alongside with decreased fruit decay throughout storage [4,23]. Moreover, Santoro et al. [26] reported that vapor application of thyme and savory EOs on peaches and nectarines was found to improve fruit’s quality attributes (i.e., less weight loss and no significant losses of ascorbic acid and carotenoid content), but at the same time they showed conflicting results on postharvest diseases (brown rot and gray mold). Among EOs, rosemary and eucalyptus have been studied for their many beneficial properties and many uses have been proposed [27,28].

Even though EOs are classified as generally recognized as safe (GRAS) food additives, it is noteworthy that their application might result in phytotoxicity, allergies and undesired alterations in product quality (i.e., appearance, aroma, flavor) if used with inappropriate (high) concentrations and/or food combinations [18,19,29]. Thus, the aim of this study was to evaluate the effects of an eco-product (EP—based on rosemary and eucalyptus essential oils) by two different application methods (vapor and dipping) on the quality attributes of tomato fruits throughout storage at 11 °C and 90% relative humidity for 14 days.

## 2. Results

### 2.1. Preliminary Test

**Figure 1:**
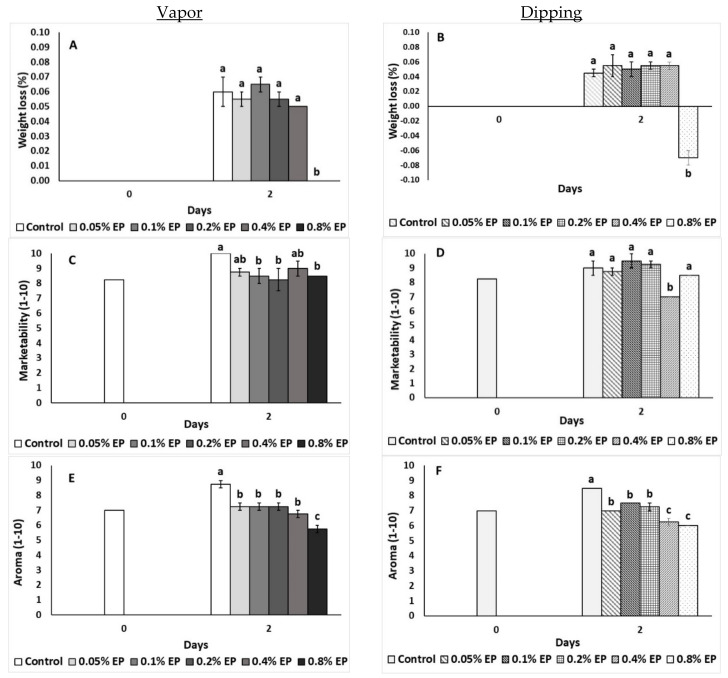
Effects of vapor (**A**,**C**,**E**) or dipping (**B**,**D**,**F**) application with eco-product (EP) at different concentrations (0%, 0.05%, 0.1%, 0.2%, 0.4% and 0.8%) or control (application of water) on weight loss (%), marketability (scale 1–10) and aroma (scale 1–10) of tomato fruits stored for two days at 11 °C. In each day, means (±SE) followed by different Latin letters significantly differ according to Duncan’s MRT (*p* = 0.05).

The effects of the EP on tomato during the preliminary screening are shown in Figure 1. Both application methods (vapor and dipping) at the highest concentration (0.8% EP) resulted in decreased weight loss compared to the other concentrations tested (Figure 1A,B). Vapor application led to lower scoring on the marketability scale with 0.1%, 0.2% and 0.8% EP, whilst dipping application with 0.4% EP also presented lower scores after two days of storage (Figure 1C,D). Furthermore, all applied concentrations (for both application methods) showed lower scoring values on the aroma scale compare with the control. However, all tested concentrations showed higher scores as compared to the higher applied concentrations (i.e., for vapor: 0.8% and for dipping: 0.4% and 0.8%) (Figure 1E,F).

### 2.2. Main Experiment

#### 2.2.1. Weight Loss and Decay

**Figure 2:**
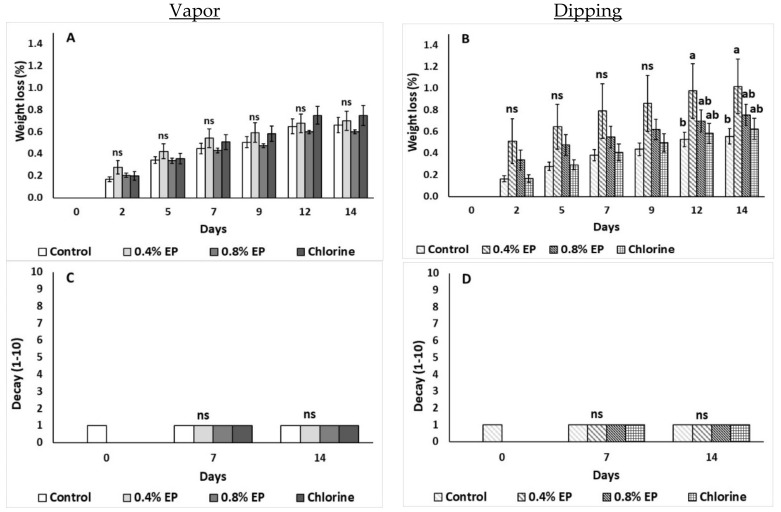
Effects of vapor (**A**,**C**) or dipping (**B**,**D**) application with eco-product (EP) at different concentrations (0%, 0.4% and 0.8%), chlorine (0.02%) or control (application of water) on weight loss (%) and decay (scale 1–10) of tomato fruits stored up to 14 days at 11 °C. In each day, means (±SE) followed by different Latin letters significantly differ according to Duncan’s MRT (*p* = 0.05). ns: not significant.

Figure 2 illustrates the effects of the EP application (vapor and dipping) on the weight loss and decay of tomato fruits. Dipping application with 0.4% EP resulted in increased weight loss on the 12th day and up to the last day of storage (0.98 and 1.02%, respectively) (Figure 2B), while no differences on weight loss were observed after vapor application (Figure 2A). No significant differences (*p* > 0.05) regarding the decay of tomato fruits were reported throughout storage for both application methods (Figure 2C,D).

#### 2.2.2. Respiration Rate and Ethylene Production

**Figure 3:**
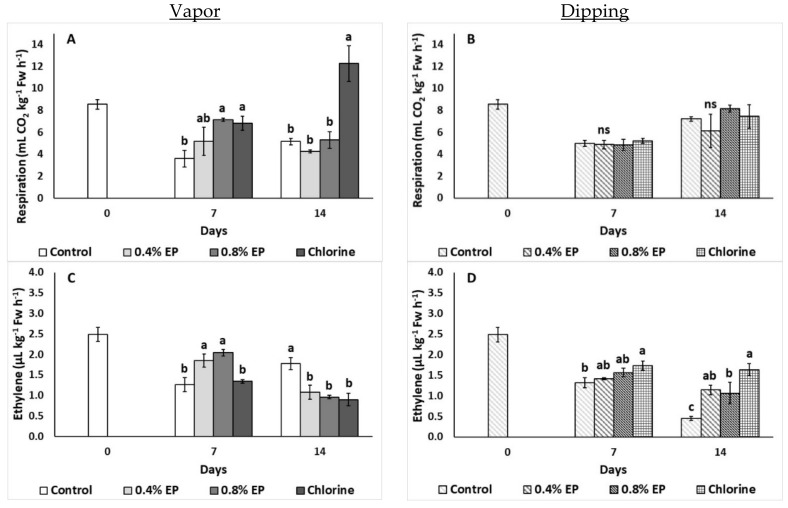
Effects of vapor (**A**,**C**) or dipping (**B**,**D**) application with eco-product (EP) at different concentrations (0%, 0.4% and 0.8%), chlorine (0.02%) or control (application of water) on respiration rate (mL CO_2_ kg^−1^ Fw h^−1^) and ethylene production (μL kg^−1^ Fw h^−1^) of tomato fruits stored up to 14 days at 11 °C. In each day, means (±SE) followed by different Latin letters significantly differ according to Duncan’s MRT (*p* = 0.05). ns: not significant.

Figure 3 presents the effects of the EP application (vapor and dipping) on tomato’s respiration rate and ethylene production. The vapor application of 0.8% EP and chlorine treatment increased respiration rate on the seventh day of storage (7.14 mL CO_2_ kg^−1^ h^−1^) and this was evident for the chlorine application even at the last day of storage (Figure 3A). Interestingly, dipping application did not significantly affect tomato’s respiration rate (*p* > 0.05) (Figure 3B). Ethylene production was increased on the 7th day of storage with EP (0.4% and 0.8%) vapor application, but this was not persistent after 14 days of storage (Figure 3C). Indeed, dipping application with chlorine increased ethylene production on the 7th day, while both EP and chlorine had increased ethylene levels on the last day of storage, compared to the control (Figure 3D).

#### 2.2.3. Firmness, Total Soluble Solids, Titratable Acidity and Ripening Index

**Figure 4:**
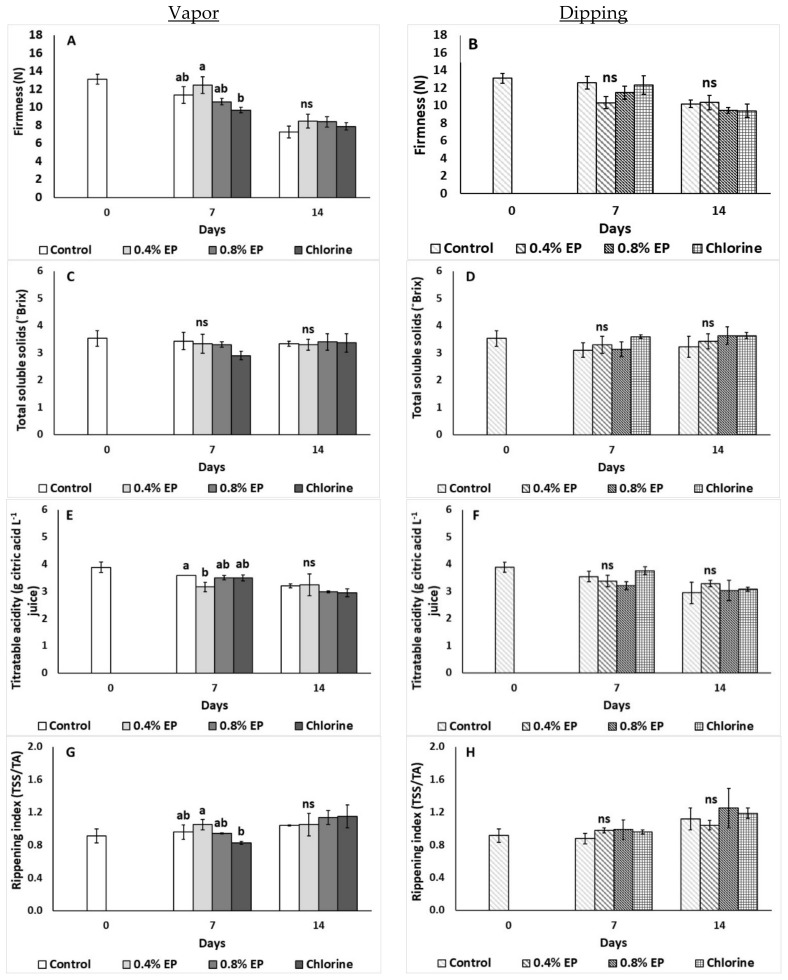
Effects of vapor (**A**,**C**,**E**,**G**) or dipping (**B**,**D**,**F**,**H**) application with eco-product (EP) at different concentrations (0%, 0.4% and 0.8%), chlorine (0.02%) or control (application of water) on firmness (N), total soluble solids (TSS; °Brix), titratable acidity (TA; g citric acid L^−1^ juice) and ripening index (TSS/TA) of tomato fruits stored up to 14 days at 11 °C. In each day, means (±SE) followed by different Latin letters significantly differ according to Duncan’s MRT (*p* = 0.05). ns: not significant.

The effects of the EP on tomato’s quality attributes (firmness, TSS, TA, ripening index) are presented in Figure 4. Fruit firmness was maintained with the EP vapor application compared to control treatment, while chlorine in vapors, decreased firmness compared to 0.4% EP treatment. Fruit firmness was maintained in similar levels after all dipping applications (Figure 4A,B). Interestingly, both methods did not significantly affect TSS of tomato fruit (Figure 4C,D). Vapor treatment of 0.4% EP decreased TA on the 7th day of storage (0.16 g citric acid L^−1^), while chlorine applied via vapor decreased fruit’s ripening index compared to 0.4% EP on the same day (Figure 4E,G). Dipping application resulted in no differences on tomato’s TA and ripening index (Figure 4F,H).

#### 2.2.4. Ascorbic Acid, Lycopene, β-carotene

**Figure 5:**
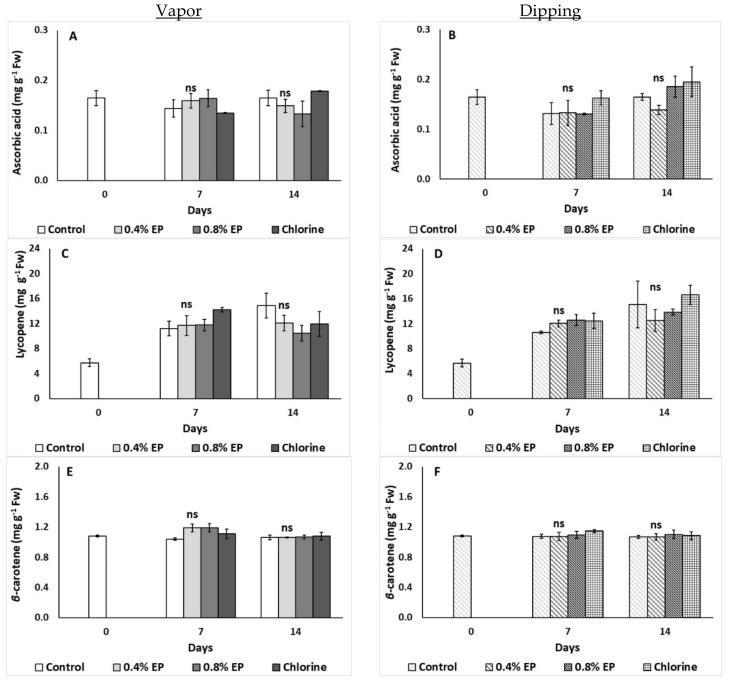
Effects of vapor (**A**,**C**,**E**) or dipping (**B**,**D**,**F**) application with eco-product (EP) at different concentrations (0%, 0.4% and 0.8%), chlorine (0.02%) or control (application of water) on ascorbic acid (mg g^−1^ Fw), lycopene (mg g^−1^ Fw) and *β*-carotene (mg g^−1^ Fw) of tomato fruits stored up to 14 days at 11 °C. In each day, means (±SE) followed by different Latin letters significantly differ according to Duncan’s MRT (*p* = 0.05). ns: not significant.

Figure 5 shows the effects of vapor and dipping application of EP and chlorine on ascorbic acid, lycopene and *β*-carotene content of tomato fruits. It is noteworthy that tomato’s ascorbic acid, lycopene and *β*-carotene contents did not significantly differ among treatments for both application methods (vapor and dipping) (*p* > 0.05) (Figure 5A–F).

#### 2.2.5. Total Phenolic Content and Antioxidant Activity

**Figure 6:**
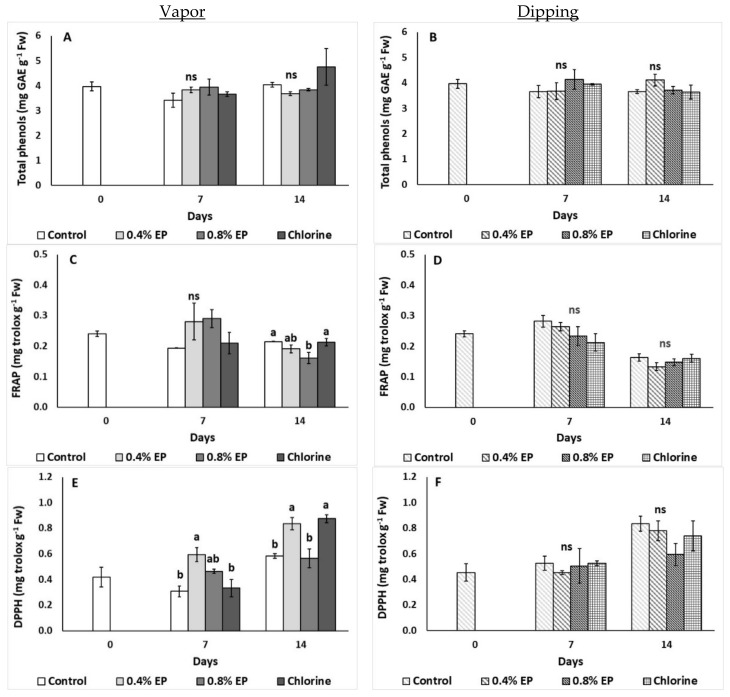
Effects of vapor (**A**,**C**,**E**) or dipping (**B**,**D**,**F**) application with eco-product (EP) at different concentrations (0%, 0.4% and 0.8%), chlorine (0.02%) or control (application of water) on total phenols content (mg GAE g^−1^ Fw) and antioxidant activity (FRAP, DPPH; mg trolox g^−1^ Fw) of tomato fruits stored up to 14 days at 11 °C. In each day, means (±SE) followed by different Latin letters significantly differ according to Duncan’s MRT (*p* = 0.05). ns: not significant.

The effects of vapor and dipping application of EP and chlorine on total phenolic content and antioxidant activity (FRAP, DPPH) of tomato fruit are presented in Figure 6. No significant differences were reported on tomato’s total phenolic content among treatments for both application methods (vapor and dipping) (*p* > 0.05) (Figure 6A,B). Vapor application of 0.8% EP decreased antioxidant activity on the last day of storage (FRAP: 0.16 mg trolox g^−1^ Fw) compared to control and chlorine treated fruits (0.22 and 0.21 mg trolox g^−1^ Fw, respectively) (Figure 6C). Antioxidant activity (DPPH) of tomato fruit increased when fruit treated with 0.4% EP on the seventh day (0.60 mg trolox g^−1^ Fw), while vapor application of 0.4% EP and chlorine also increased antioxidants on the last day of storage (0.84 and 0.88 mg trolox g^−1^ Fw, respectively) (Figure 6E). Interestingly, dipping application resulted in no differences on tomato’s antioxidant activity (assayed by FRAP and DPPH) (Figure 6D,F).

#### 2.2.6. Damage Index

**Figure 7:**
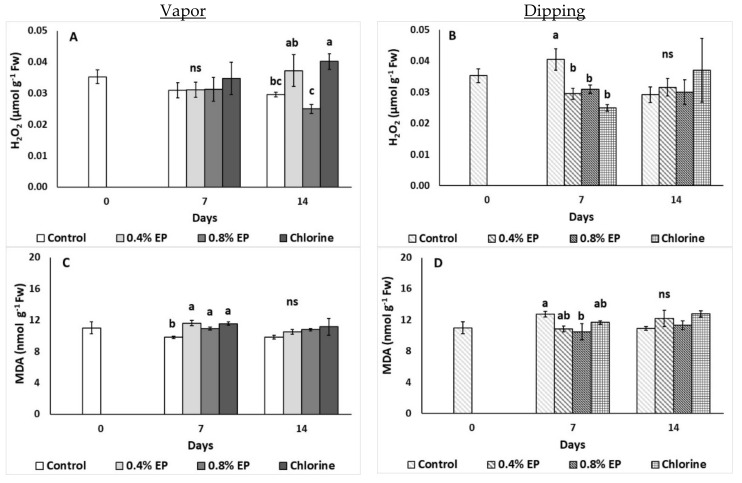
Effects of vapor (**A**,**C**) or dipping (**B**,**D**) application with eco-product (EP) at different concentrations (0%, 0.4% and 0.8%), chlorine (0.02%) or control (application of water) on damage index of tomato fruits (H_2_O_2_ and MDA levels; μmol g^−1^ Fw and nmol g^−1^ Fw, respectively) stored up to 14 days at 11 °C. In each day, means (±SE) followed by different Latin letters significantly differ according to Duncan’s MRT (*p* = 0.05). ns: not significant.

The effects of the EP application on the damage index of tomato fruit (H_2_O_2_ and lipid peroxidation levels) are illustrated in Figure 7. The vapor application of chlorine increased H_2_O_2_ levels of tomato compared to 0.8% EP-treated and control fruits (0.03 and 0.03 μmol g^−1^ Fw, respectively) on the last day of storage (Figure 7A). On the other hand, all treatments applied via dipping decreased tomato’s H_2_O_2_ levels on the seventh day, compared to control fruits (Figure 7B). All vapor applied treatments resulted in increased lipid peroxidation (increased MDA levels) during the seventh day (compared to untreated fruits). Dipping tomato fruits in 0.8% EP lowered MDA levels in comparison with control fruits (11.34 and 10.90 nmol MDA g^−1^ Fw, respectively) on the 14th day (Figure 7C,D).

#### 2.2.7. Enzymes Antioxidant Activity

**Figure 8:**
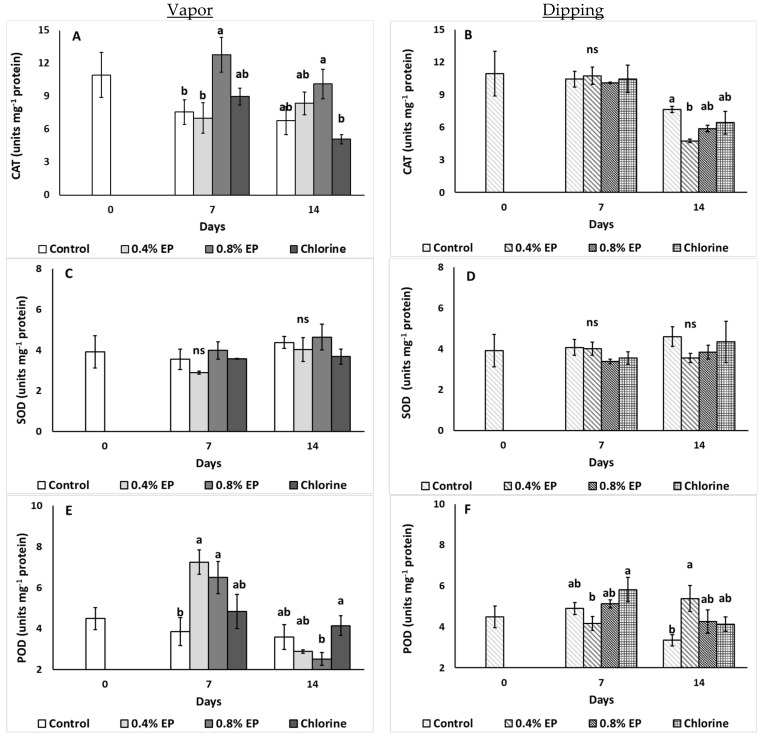
Effects of vapor (**A**,**C**,**E**) or dipping (**B**,**D**,**F**) application with eco-product (EP) at different concentrations (0%, 0.4% and 0.8%), chlorine (0.02%) or control (application of water) on enzyme activity (catalase-CAT, superoxide dismutase-SOD and peroxidase-POD), expressed as units of enzyme per mg of protein, stored up to 14 days at 11 °C. In each day, means (±SE) followed by different Latin letters significantly differ according to Duncan’s MRT (*p* = 0.05). ns: not significant.

Figure 8 illustrates the effects of the EP application (vapor and dipping) on the enzymatic activity of tomato fruits. During the seventh day of vapor application of 0.8% EP, CAT activity was increased (12.78 units mg^−1^ protein) compared to control and 0.4% EP (7.55 and 7.01 units mg^−1^ protein, respectively), while 0.8% EP also increased CAT activity on the last day of storage in comparison to chlorine treated fruits (Figure 8A). On the other hand, dipping in 0.4% EP decreased CAT activity on the 14th day compared to control (Figure 8B). Interestingly, both EP application methods did not significantly affect SOD activity of tomato fruits (Figure 8C,D). POD activity increased with the EP vapor application (0.4 and 0.8%) compared to control on the seventh day of storage, while 0.8% EP led to decreased enzyme activity on the last day of storage compared to chlorine (2.52 and 3.59 units mg^−1^ protein, respectively) (Figure 8E). In contrast, dipping in 0.4% EP resulted in decreased POD activity on the seventh day of storage compared to chlorine, whilst the same treatment increased enzymes’ activity on the last day of storage in comparison to control (Figure 8F).

#### 2.2.8. Sensory Evaluation

**Figure 9:**
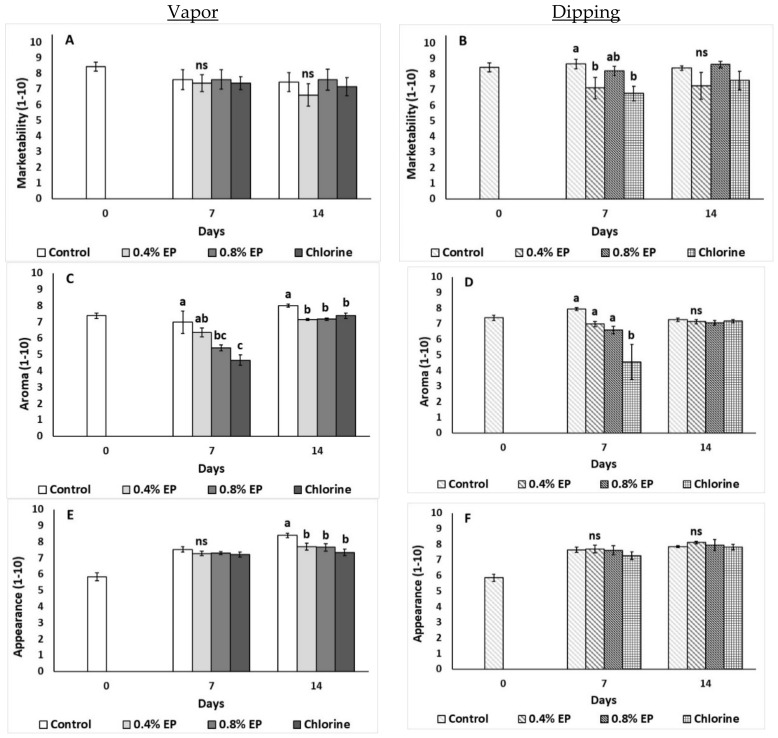
Effects of vapor (**A**,**C**,**E**) or dipping (**B**,**D**,**F**) application with eco-product (EP) at different concentrations (0%, 0.4% and 0.8%), chlorine (0.02%) or control (application of water) on marketability (scale 1–10), aroma (scale 1–10) and appearance (scale 1–10) of tomato fruits stored up to 14 days at 11 °C. In each day, means (±SE) followed by different Latin letters significantly differ according to Duncan’s MRT (*p* = 0.05). ns: not significant.

The effects of the EP’s application (via vapor and dipping) on tomato’s sensory attributes (marketability, aroma, appearance) are illustrated in Figure 9. Tomato’s marketability was not affected during vapor application (Figure 9A), while dipping on 0.4% EP and chlorine decreased marketability (lower scoring values) on the seventh day of storage compared to control (Figure 9B). Vapor treatment with chlorine decreased aroma scoring on the seventh day of storage compared to control, while on the last day of storage all vapor treatments were able to decrease aroma values (Figure 9C). Moreover, dipping in chlorine resulted in lower aroma values on the seventh day compared to other treatments (Figure 9D). During vapor application, all treatments resulted in lower appearance values compared to control on the last day of storage, while dipping method did not affect tomato’s appearance (Figure 9E,F).

## 3. Discussion

Tomato is an important food crop, characterized by high consumption numbers worldwide, and numerous uses and health benefits [30]. As a fresh produce commodity, tomato has a relatively short shelf life and its quality is affected by many pre and postharvest factors. During postharvest handling many parameters can influence tomato’s quality attributes resulting in rapid deterioration. In this study, dipping tomato fruits in 0.4% EP increased product’s weight loss after the 12th day of storage; however, the weight loss was less than 1.2%, which is not considered of great issue in postharvest storability of fresh produce. Similarly, Tzortzakis et al. [23] reported increased weight loss with dipping tomatoes in 0.5% sage EO compared to 0.1% EO and control fruits after seven and 14 days of storage at 11 °C. In another study, the vapor application of oregano EO did not result in any significant differences in tomato’s weight loss [31]. These effects can be attributed to the similar EO composition of the main components of rosemary (isoborneol, α-pinene, α terpineol, 1.8-cineole), eucalyptus (1.8-cineole, α-pinene and δ-3 carene) and sage (α-thujone, camphor, 1.8-cineole, camphene and α-pinene) in comparison to oregano EO (carvacrol, *p*-cymene, γ-terpinene) [4,23,31,32].

Tomato, as a typical climacteric fruit, is characterized by increased respiration rate followed by increased ethylene production while ripening [33]. In that sense, vapor application increased respiration rates mainly at high EP levels and chlorine after seven days and at chlorine application at 14 days. Such changes in respiration were not evidenced in dipping application, indicating a non-lasting impact of the dipping, compared to the vapor method. We speculate that any boost in respiration rates of dipped tomatoes could possibly happen before the seven days, as indicated by the increased ethylene levels at days 7 and 14. However, considering the effects of EP product, vapor application of EP (0.4%, 0.8%) increased ethylene emission on the seventh day, while respiration increase was observed only at the 0.8% EP vapor application, on the same day. Both vapor and dipping application methods retarded ethylene emission on the last day of storage compared to day zero (fruits were at room temperature), and this is evidenced by the low temperatures used in the present study in comparison to room temperature, by retarding the ripening process and slowing down metabolic changes of the fruit. In a previous study, it has been shown that respiration rate and ethylene emission of tomato fruit were increased after dipping the fruits in 0.5% sage EO, compared to control and 0.1% EO, after the seventh day and up to the last day (14th) of storage at 11 °C [23]. The differences in respiration rate and ethylene emission of these products can be attributed to disturbance of gas exchange and cell wall degradation that can be caused by the duration of the EO application and/or even the method of application [34,35]. The increased respiration and/or ethylene production are processes related to increased fruit metabolism and ripening.

Tomato fruit firmness decreases through storage and maturation as fruit gets softer, while it has been previously suggested that EO treated fruits maintained higher firmness values compared to non-treated fruits [35]. Oregano EO when applied to tomato fruits was found not to affect fruit’s firmness in comparison with control fruits while at the same time it decreased fruit decay after 14 days of storage [31], highlighting a prolonged postharvest storage period. Vapor application of EP (0.4%) resulted in firmness maintenance compared to vapor chlorine on the seventh day of storage. Moreover, in the present study, no symptoms of decay were observed on the examined tomatoes in all treatments and methods of applications (vapor vs. dipping). Similarly, in another study, eucalyptus EO vapor-treated tomatoes maintained their firmness [36]. No significant differences regarding firmness were reported with the application (via dipping) of sage EO (0.5% and 0.1%) on tomato fruits even up to 14 days of storage at 11 °C [23]. These results are in accordance with the observations of dipping application of the EP (0.4% and 0.8%) in the present study. Indeed, EO effectiveness from the previous mentioned EOs on fruit firmness is attributed to the common main components of the tested EOs, such as 1,8-cineole and α-pinene; however, that statement needs further substantiation by testing individual chemical components and/or mixtures of them in ratios similar to the examined EOs. The Eos’ effectiveness is not related only to the main component of the oil, but to the synergistic action of the major components, usually numbering 3–5 components in each EO. Nevertheless, EOs effectiveness can vary, and that can be attributed to the different species, even varieties, to the cultivation practices, and components’ composition.

Acidity (organic acids) is one of the main taste characteristics of tomatoes that influence fruit quality and decreases throughout storage, as fruit ripens [37,38]. Tomato’s TA was decreased on the seventh day by the vapor application of 0.4% EP. The use of sage EO decreased tomato’s TA, while increasing its sweetness (ripening index) after 14 days of storage at 11 °C [23]. Similarly, Adams et al. [35] mentioned a decrease in TA in EO-treated (ginger EO) tomatoes compared to control. Aminifarda and Mohammadi [39] reported that tomato fruits treated (dipping method) with ammi and anise EOs (concentration range: 200–800 μL L^−1^) presented higher TSS compared to the control ones. During the ripening of tomato fruits, sugars accumulate above the required levels for respiration purposes and this is reflected in an increased TSS value [37]. In the present study, both application methods (dipping and vapor) did not affect tomato’s TSS throughout storage. However, it has been previously mentioned that EO-treated tomatoes seem to have a slightly decreased TSS due to respiratory metabolism [35].

Color is one of the most important quality attributes of tomato fruit. The development of pigments changes during tomato fruit maturation, with the production of carotenoids and the breakdown of chlorophylls. In the present study, both application methods (dipping and vapor) did not affect tomato’s AA, *β*-carotene and lycopene content throughout storage, which is important for maintaining the fruit’s high nutritive value. These results are of agreement with another study in which no significant differences were reported on the carotenoid content of peaches and nectarines with the application of thyme and savory EO (1% and 10% in vapor phase) [26]. Carotenoid content decreases during storage and exposure of fruits to light, however the use of EOs has been proven to prevent oxidation processes (scavenging free radicals) and even preserve carotenoid levels due to their antioxidant activities [8]. On the other hand, the application of 0.1% sage EO resulted in higher AA levels of tomato fruits compared to control and 0.5% EO treated fruits [23]. Interestingly, AA and carotenoid content (*β*-carotene and lycopene) were found to be increased with the dipping of tomato fruits in ammi and anise EOs (concentrations range: 200–800 μL L^−1^) compared to control fruits, initiating the antioxidative metabolism of the fruit [39]. Moreover, application of oregano EO (0.40 mL L^−1^) resulted in tomato’s increased lycopene and AA content (compared to control fruits) after 14 days of storage [31]; however, oregano EO levels were 10-fold lower than the ones used in the present study (i.e., 0.4%). Santoro et al. [26] reported that the reduced loss of the nutritional value macromolecules such as AA and carotenoids might be attributed to the EOs and their components (i.e., 1,8-cineole, α-pinene and camphor) that possess antioxidant activities, protecting sensitive nutrients from oxidation.

The results of this study indicate that both application methods (dipping and vapor) did not cause significant changes in total phenolic content. Considering that AA, carotenoids (*β*-carotene and lycopene), and total phenolics remained unchanged in general, this indicates that non-enzymatic antioxidants compounds were not influenced by the examined treatments. Therefore, any possible stress, as indicated by MDA and H_2_O_2_ increases, could alter the enzymatic antioxidant capacity of the fruit (for example, the increased CAT and POD values). Tzortzakis et al. [23] also reported no significant differences in phenolic content of tomato fruits treated with sage EO (0.1% and 0.5% via dipping). Additionally, in the same study no significant differences in antioxidant levels (DPPH, FRAP) were observed on the seventh day of storage, while increased antioxidants were reported on the last day of storage when tomato fruits were dipped in 0.1% sage EO (compared to 0.5% EO and control) [23], being in agreement with our results of 0.4% EP (vapor method) for DPPH activity at seven days of storage. The application of EOs on fresh produce can enhance the content as well as the production of antioxidant compounds when applied to fresh produce due to their own antioxidant capacity [23]. However, the increase in antioxidants during fresh produce storage is also attributed to other extrinsic postharvest factors such as chilling or adverse storage temperatures and low RH, among others.

Considering damage indices, dipping in EP product decreased tomato’s H_2_O_2_ levels on the seventh day of storage, whereas dipping in 0.8% EP decreased MDA levels on the same day. The decreased MDA levels observed in EO-treated fruits can be attributed to the induced activation of defense-related enzymes, towards the oxidative stress challenge [40]. When citrus fruits were treated with 0.5% clove EO, increased H_2_O_2_ levels were revealed up to four days of storage at 25 °C [40]. At the same study, clove EO-treated fruits presented lower MDA levels compared to non-treated fruits from 12 h after the application and up to four days [40]. Another study mentioned that kiwifruits treated with 0.6 μL mL^−1^ citral presented decreased H_2_O_2_ and MDA levels when stored at 1 °C for up to 90 days [41]. On the other hand, our findings indicate that vapor application of the EP increased MDA levels (increased lipid peroxidation) during the seventh day of storage. Shao et al. [42] reported that vapor application of tea tree EO (0.9 g L^−1^) increased H_2_O_2_ levels of strawberry fruits stored at 20 °C for three days. Increased oxidative stress—production of reactive oxygen species (ROS) such as H_2_O_2_—can be reported throughout storage of fresh produce and this might be attributed to the ripening process, the time and conditions of storage as well as the applied treatments (including the application of EOs). EOs when applied in fruits seem to directly and indirectly initiate the activation of fruit defense mechanisms, including the activity of antioxidant enzymes [42].

It has been previously mentioned that the application of clove EO (0.4%) on citrus fruits enhanced the activity of plant defense-related enzymes including POD, phenylalanine ammonia-lyase (PAL), polyphenol oxidase (PPO), and lipoxygenase (LOX) [40]. In the present study, the application of EP (based on rosemary and eucalyptus essential oil components) revealed an increase in the CAT and POD activity of tomato’s fruits during the seventh day of storage, highlighting the induction of enzymatic antioxidant mechanisms of the fruit. Similarly, tea tree EO vapor treated (0.9 g L^−1^) strawberries showed higher SOD, PAL and POD activities during a three-day storage at 20 °C [42]. In our study, a decrease in POD activity was reported on the seventh day with dipping application of 0.4% EP, followed by an increase in POD activity on the 14th day. These results differ from a previous study where increased POD activity was recorded throughout storage of citral-treated (0.6 μL mL^−1^) kiwifruits [41], and such EO variation in its effectiveness is related to the EO composition, application practices and/or tested commodities. However, both tomatoes and kiwifruits are climacteric fruits and a speed-up of the ripening metabolism is taking place during storage, as indicated by the increased respiration and ethylene emission rates, mainly after seven days of storage. EO-treated fruits tend to present increased activity of antioxidant enzymes in order to suppress the accumulation of ROS during ripening and senescence of fruits during storage conditions and processing. Moreover, the activity of POD has also been correlated with fresh produce quality attributes (including color) [8]. This might also partially explain the lower appearance scoring values of tomato fruits vapor treated with the EP.

Sensory evaluation (aroma, optical/visual appearance) of fresh produce is one of the main factors affecting consumers’ buying decisions. In the present study, dipping tomato fruits in 0.4% EP decreased marketability on the seventh day compared to control fruits. In contrast, the application of ginger EO (application time 20 and 30 min) increased acceptance of tomato fruit compared to control fruits [35]. Furthermore, the application of 0.8% EP (vapor method) in our study resulted in a less tomato-like aroma on the seventh day, while all applied vapor treatments also decreased aroma (less tomato-like aroma) on the last day of storage. When fruits were treated with the vapor method (all treatments), the appearance of the fruits decreased on the last day of storage indicating a less red product (less acceptable by consumers). Tzortzakis et al. [23] mentioned that tomato fruits treated with 0.1% sage EO presented greater appearance, aroma, texture and marketability compared to control fruits. On the other hand, the same study reports that increased sage EO concentration (0.5%) resulted in a less acceptable product [23]. The time of application can also affect the quality of the end product. In a previous study, the dipping application of ginger EO for 20 min resulted in a less acceptable (“sour”) product compared to 30 min application which resulted in a more acceptable product [35]. Further investigation of EO and EP applied to postharvest fresh produce is needed, as it is essential that fresh produce and applied EOs are being combined in a harmonious manner enhancing fresh produce quality attributes.

## 4. Materials and Methods

### 4.1. Plant Material and Experimental Set Up

The present study took place at the laboratory facilities of Food Science and Technology of the Cyprus University of Technology, Limassol, Cyprus. Tomato (*Solanum lycopersicum* cv. F179) fresh produce were purchased from a commercial greenhouse. Crops were grown under common cultivation practices in a clay sandy-loam soil and drip irrigation and fertigation were applied according to crop needs. The cultivation took place during winter–spring months and the temperature ranged between 19 °C and 31 °C.

An eco-product (EP; named as ‘’Agriculture Green-tech E’’, Meydan Solution Ltd., Larnaca, Cyprus) based on rosemary (*Rosmarinus officinalis* L.) and eucalyptus (*Eucalyptus crabra* L.) essential oil was used. Individual EOs were analyzed by gas chromatography-mass spectrometry (GC/MS-Shimadzu GC2010 gas chromatograph interfaced Shimadzu GC/MS QP2010 plus mass spectrometer) and constituents were determined [43] and presented in Table S1. *Rosemarinus officinalis* essential oils used in the tested formula were found rich in isoborneol (30.29%), α pinene (25.71%), α terpineol (14.89%) and 1.8-cineole (10.81%), while the dominant compounds of the essential oils from *Eucalyptus crabra* were 1.8-cineole (26.51%), α pinene (24.12%) and δ-3 carene (20.10%), being in accordance with previous studies [44,45]. This product was a mixture of these two essential oils (eucalyptus: rosemary in approximately 2:1 *v*/*v* ratio) and it also contained vinegar < 5% *w*/*w* as well as emulsifier treated water (<80%). Chlorine was used as a commercial sanitizer at 0.02% (*v*/*v*).

Fresh produce was selected based on uniform size, appearance, and absence of physical defects and used immediately in the different experiments. The fruits were disinfected with a chlorine (0.05% *v*/*v*) solution for 4 min and washed four times with distilled water before use (to avoid any microbial load). A preliminary screening and main experiments were implemented.

### 4.2. Preliminary Test

A preliminary experimental set-up has been conducted in order to determine the possible phytotoxicity or negative effects of the EP on the examined produce quality. Tomatoes were placed in 1 L capacity polystyrene (2 fruits per container), snap-on lid containers.

A total of five concentrations of the EP were examined (0.05%–0.1%–0.2%–0.4%–0.8% *v*/*v*), while purified water (0.0% EP) was used as control treatment and applied either as vapor or dipping. Three replications were used for each concentration and for each application. Containers were placed in a chamber at 11 °C and 90% relatively humidity (RH), in the dark. To maintain high RH during storage period, wet filter paper was displaced inside each container, and was remoistened every second day [46]. Container lids were open every 48 h and aerated in order to avoid air composition abnormalities (i.e., decreased O_2_ and increased CO_2_ levels). Fresh produce was monitored for phytotoxicity (marked spots), marketability, aroma and weight loss (as described at the main experiment) after 2 days of storage. Based on the results, the concertation of 0.4% was selected for further investigation and it was compared with a double level of the eco-product (0.8% EP) and common postharvest sanitizer (i.e., chlorine) in vapor or dipping applications.

### 4.3. Main Experiment

The following four treatments were examined: (i) purified water (control), (ii) 0.4% EP, (iii) 0.8% EP (iv) chlorine (0.02%). The treatments were applied either in vapor or dipping. For vapor application, tomatoes were placed in the container, together with the vaporized solution of the selected concentrations (in a 2 mL Eppendorf tube). For dipping application, based on the preliminary tests and previous studies [8], fresh produce was immersed for 10 min in the examined solution and then fruits were left to dry for 20 min at room temperature. Then, each two tomatoes were placed into the polystyrene, snap-on lid containers (1 L capacity). In total, six biological replications per treatment, for each storage period of 7 and 14 days, were used. Containers were placed in a refrigerated chamber at 11 °C and 90% relatively humidity (RH), in the dark. In summary, the experimental set up consisted of four treatments × six replications × two storage periods (plus day 0) and two application (dipping and vapor) methods. Fresh produce enclosed in containers was kept at room temperature for 2–3 h to allow EP vapor-activation, and then transferred to chilled conditions. To maintain high RH during storage period, wet filter paper was displaced inside each container, and was remoistened every second day, as described above. Container lids were open every 48 h and aerated in order to avoid air composition abnormalities.

#### 4.3.1. Decay Evaluation

Fruit decay was visually evaluated at days 7 and 14, after storage at 11 °C. All fresh produce from each container were used for the evaluation. A commodity was considered as decayed when the symptoms of mycelia or bacteria development were present. A scale from 1 to 10 showing the surface infection percentage as 1: 0–10% infection; 2: 11–20% infection; 3: 21–30% infection; 4: 31–40% infection; 5: 41–50% infection; 6: 51–60% infection; 7: 61–70% infection; 8: 71–80% infection; 9: 81–90% infection; and 10: 91–100% infection was assessed to estimate the degree of produce infection.

#### 4.3.2. Respiration Rate and Ethylene Production

The carbon dioxide (CO_2_) and ethylene production were assessed by placing each fruit in a 1 L plastic container sealed for 1 h, at room temperature. Each fruit was weighed and its volume was measured. Moreover, CO_2_ and ethylene of room air were tested and subtracted from the measurements, by equipment zeroing, prior to and during measurement. For the determination of the respiration rate, container gas atmosphere was sucked by a dual gas analyzer (GCS 250 Analyzer, International Control Analyser Ltd., Kent, UK) for 40 s and results were expressed as milliliter of CO_2_ per kilogram per hour (mL CO_2_ kg^−1^ h^−1^). Ethylene was measured with an ethylene analyzer (ICA 56 Analyzer, International Control Analyser Ltd., Kent, UK) whereas the container air sample was sucked for 15 s. Results were expressed as microliter of ethylene per kilogram per h (μL ethylene kg^−1^ h^−1^) (three replications per treatment and storage period; *n* = 3).

#### 4.3.3. Weight Loss and Fruit Firmness

Fruit weight was recorded on the harvesting day (day 0) for each fresh produce (*n* = 6) per treatment and every other day, up to the last day of storage (day 14). Fruit firmness was measured at two points on the shoulder of each tomato fruit by applying a plunger of 3 mm in diameter at a speed of 2 mm s^−1^ and the penetration depth was 12 mm, using a texture analyzer (TA.XT plus, Stable Micro Systems, Surrey, UK). The amount of force (in Newtons; N) required to break the radial pericarp (i.e., surface) of each commodity (*n* = 6) was recorded at ambient temperature (22–24 °C).

#### 4.3.4. Soluble Solids, Titratable Acidity, Ascorbic Acid and Carotenoids

Total soluble solids (TSS) concentration was determined in triplicate from the juice obtained from two pooled fresh fruits for each replication (*n* = 3) with a temperature-compensated digital refractometer (model Sper Scientific 300017, Scottsdale, AZ, USA) at 20 °C, and results were expressed in °Brix. Titratable acidity (TA) was measured via potentiometric titration (Mettler Toledo DL22, Columbus, OH, USA) of 5 mL of fruit juice diluted to 50 mL with distilled water, using 0.1 N NaOH up to pH 8.1. Results were expressed as g of citric acid per 1 L juice (g citric acid L^−1^ juice). The fruit sweetness/ripening index was calculated using TSS/TA ratio.

Ascorbic acid (AA) was determined by the 2,6-Dichloroindophenol titrimetric method as described previously [47]. An aliquot of 5 mL of pooled tomato juice was diluted with 45 mL of oxalic acid 0.1% and was titrated by the dye solution until the color changed. Data were expressed as mg of AA per gram of fresh weight (mg AA g^−1^ Fw).

Carotenoids (lycopene and *β*-carotene) for tomatoes were determined according to the method described by Nagata and Yamashita [48]. Briefly, 1 g of blended tomatoes was homogenized with 20 mL of acetone:hexane 4:6 (*v:v*) and after sonication and vigorous vortex the two phases were separated automatically. An aliquot from the upper phase was used for absorbance measurement at 663, 645, 505 and 453 nm in a spectrophotometer, using a reference of acetone:hexane (4:6) ratio. Lycopene and *β*-carotene contents were calculated according to the Nagata and Yamashita [48] equations:

Lycopene (mg 100 mL^−1^ of extract) = −0.0458 × A_663_ + 0.204 × A_645_ + 0.372 × A_505_ − 0.0806 × A_453_. (1)

*β*-Carotene (mg 100 mL^−1^ of extract) = 0.216 × A_663_ − 1.22 × A_645_ − 0.304 × A_505_ + 0.452 × A_453_. (2)

Results were expressed as mg per gram of fresh weight (mg g^−1^ Fw).

#### 4.3.5. Total Phenolics and Antioxidant Activity

Total phenolic content was measured in blended fruit tissue (1 g) extracted with 10 mL of 50% (*v*/*v*) methanol, as reported previously [43]. Results were expressed as mg gallic acid equivalents (GAE) per gram of fresh weight (mg GAE g^−1^ Fw). The antioxidant activity was determined using the ferric-reducing antioxidant power (FRAP) and 2,2-diphenyl-1-picrylhydrazyl (DPPH) radical-scavenging activity assays (at 593 and 517 nm, respectively) as described by Chrysargyris et al. [43]. The results were expressed in mg trolox per gram of fresh weight (mg trolox g^−1^ Fw). All biological samples were analyzed in triplicate.

#### 4.3.6. Damage Index (Hydrogen Peroxide and Lipid Peroxidation)

Hydrogen peroxide (H_2_O_2_) levels were estimated using the procedure previously described by Loreto and Velikova [49]. After measuring the optical density (OD) at 390 nm, results were expressed as μmol of H_2_O_2_ per gram of fresh weight (μmol g^−1^ Fw). Lipid peroxidation was determined with the 2-thiobarbituric acid reactive substances (TBARS) assay according to de Azevedo Neto et al. [50]. The absorbance was measured at 352 nm (discarding the non-specific absorbance at 600 nm) and results were expressed as nmol malondialdehyde (MDA) per gram of fresh weight (nmol g^−1^ Fw).

#### 4.3.7. Enzymatic Antioxidant Activity

Fresh fruit tissue, powdered with liquid nitrogen (0.5 g) was homogenized with 4 mL of 50 mM potassium phosphate buffer (pH 7.0), which contained 1 mM phenylmethylsulfonyl fluoride (PMSF), 1 mM ethylenediaminetetraacetic acid (EDTA), 1% *w*/*v* polyvinylpolypyrrolidone (PVPP) and 0.05% Triton X-100. Samples were then centrifuged at 16000 g for 20 min, at 4 °C, and the supernatant was used for the determination of the antioxidant enzyme activity. Catalase (CAT, EC 1.11.1.6) activity was determined by following the consumption of H_2_O_2_ at 240 nm for 3 min. Results were expressed as CAT units per milligram of protein (units mg^−1^ protein), using the extinction coefficient of 39.4 mM cm^−1^ [51].

Superoxide dismutase (SOD, EC 1.15.1.1) was assayed as described by Chrysargyris et al. [52]. The reaction of 13 mM methionine, 75 μM nitro blue tetrazolium (NBT), 0.1 mM EDTA, 2 μM riboflavin and extract, was started after the addition of riboflavin. Reaction was then incubated under a light source of two 15-watt fluorescent lamps, for 15 min. The absorbance was determined at 560 nm and the activity was expressed as SOD units per mg of protein (units mg^−1^ protein). One unit of SOD activity was defined as the amount of enzyme required to cause 50% inhibition of the NBT photoreduction rate.

Peroxidase activity (POD, EC 1.11.1.7) was determined according to the method used by Tarchoune et al. [53], using pyrogallol as substrate. The increase in absorbance at 430 nm was measured on a kinetic cycle for 3 min, and results were expressed as units of POD per milligram of protein (units mg^−1^ protein).

An aliquot of the extract was used to determine the protein content by the Bradford method [54], with bovine serum albumin (BSA) as the protein standard.

#### 4.3.8. Sensory Evaluation

Fresh produce marketability, aroma and appearance were recorded by at least six panelists to compare the external visual aspect and marketability of treated and control fresh produce after 7 and 14 days of storage at 11 °C. Aroma was evaluated by using a 1–10 scale, with 1: bad aroma but not EP odor; 3: EP odor with some unpleasant smell; 5: EP smell but it is pleasant; 8: less tomato-like; 10: intense tomato-like. Appearance was evaluated by using a 1–10 scale, with 1: green color of 50%; 3: yellow-green; 5: orange; 8: red; 10: deep red. Marketability was evaluated by using a 1–10 scale, with 1: not marketable quality (i.e., malformation, wounds, infection); 3: low marketable with malformation; 5: marketable with few defects i.e., small size, decolorization (medium quality); 8: marketable (good quality); 10: marketable with no defects (extra quality).

### 4.4. Statistical Analysis

Statistical analysis was performed using IBM SPSS version 22 comparing data means (± standard error-SE) with one-way ANOVA, and Duncan’s multiple range tests were calculated for the significant data at *p* = 0.05. Measurements were done in three (preliminary test) or three or six (main study) biological replications/treatment (each replication consisted of a pool of two to three individual measures/sample) on different assays.

## 5. Conclusions

The EP based on the mixture of EOs (eucalyptus and rosemary) had different impacts on tomato fruits. Tomato fruit’s quality attributes including TA, ripening index as well as antioxidants were not significantly affected by EP dipping application, whilst the same application method resulted in decreased damage index (H_2_O_2_ and MDA levels). Exposure of tomato fruits to vapor EP low concentration (0.4%) increased fruit’s antioxidants. It is important to consider the application method of the EP, as it seems from this study that vapor applications had more profound effects than dipping application. The nutritional value (mostly AA and carotenoid content) remained unaffected in different EP levels and/or applications vapor and dipping). The findings of this study indicate that the investigated EP presented similar and/or better results on tomato fruits compared to those treated with chlorine (a commonly used sanitizing agent of the food industry); thus, this product could possibly be considered as an alternative and environmentally friendly agent used during postharvest handling of fresh produce. The use of natural products in fresh produce preservation should be further investigated to determine the optimum conditions of application (i.e., method, time, concentration) for each commodity examined in each case. However, caution should be taken when applying EOs and products based on their components to fresh produce commodities, since products’ sensory attributes might be adversely impacted (undesirable/intense aroma and flavor) or even allergy issues might arise during consumption.

## Data Availability

The data presented in this study are available in the article.

## Supplementary Materials

The following are available online at https://www.mdpi.com/article/10.3390/plants10061125/s1, Table S1: Chemical composition (%) of essential oils of rosemary (*Rosmarinus officinalis* L.) and eucalyptus (*Eucalyptus* *crabra* L.).
