# Supplementary material for: Quality Attributes and Storage of Tomato Fruits as Affected by an Eco-Friendly, Essential Oil-Based Product
Source: Plants (Basel). 2021 Jun 1;10(6):1125. doi: 10.3390/plants10061125 (PMC8228351; doi:10.3390/plants10061125)
Supplement: Supplementary file 1 [file plants-10-01125-s001.zip › plants-1238674-supplementary.pdf]

Article

Quality Attributes and Storage of Tomato Fruits as Affected by an Eco-friendly, Essential Oil-based Product

Panayiota Xylia, Irene Ioannou, Antonios Chrysargyris, Menelaos Stavrinides and Nikolaos Tzortzakis*

Department of Agricultural Sciences, Biotechnology and Food Science, Cyprus University of Technology, 3036, Limassol, Cyprus: pa.xylia@edu.cut.ac.cy (P.X.); i.ioannou1996@gmail.com (I.I.); a.chrysargyris@cut.ac.cy (A.C.); m.stavrinides@cut.ac.cy (M.S.)

* Correspondence: nikolaos.tzortzakis@cut.ac.cy; Tel.: +357-250-022-80

Supplementary Materials

Table S1. Chemical composition (%) of essential oils of rosemary (*Rosmarinus officinalis* L.) and eucalyptus (*Eucalyptus crabra* L.).

Rosemary				Eucalyptus			
**RI**	**Compound**	**Mean**	**SE**	**RI**	**Compound**	**Mean**	**SE**
921	tricyclene	0.44	±0.02	921	tricyclene	0.11	±0.00
926	α thujene	0.04	±0.00	926	α thujene	0.05	±0.00
933	α pinene	25.71	±1.36	933	α pinene	24.12	±0.02
948	camphene	2.88	±0.08	944	α fenchene	0.31	±0.01
977	β pinene	2.55	±0.02	948	camphene	1.37	±0.00
989	β myrcene	0.12	±0.01	977	β pinene	1.09	±0.01
1024	p cymene	1.12	±0.02	989	β myrcene	0.61	±0.01
1028	D-limonene	2.87	±0.06	1000	δ 2 carene	0.09	±0.00
1031	eucalyptol	10.81	±0.14	1004	α phellandrene	0.56	±0.00
1058	γ terpinene	0.39	±0.02	1012	δ 3 carene	20.10	±0.05
1132	terpineol-1	0.11	±0.01	1013	1,4-cineole	1.72	±0.03
1145	camphor	0.86	±0.05	1014	α terpinene	0.49	±0.04
1155	isoborneol	30.29	±0.43	1024	p cymene	1.08	±0.01
1161	trans β terpineol	0.28	±0.01	1028	D-limonene	3.82	±0.02
1166	borneol	0.37	±0.02	1031	eucalyptol	26.51	±0.06
1178	terpinen-4-ol	0.57	±0.04	1058	γ terpinene	0.69	±0.00
1191	α terpineol	14.89	±0.36	1089	terpinolene	2.48	±0.00
1196	γ terpineol	2.65	±0.11	1145	camphor	5.58	±0.03
1253	geraniol	0.16	±0.03	1155	isoborneol	0.31	±0.02
1255	linalool acetate	0.12	±0.00	1191	α terpineol	3.45	±0.03
1284	isobornyl acetate	0.87	±0.05	1196	γ terpineol	0.45	±0.01
1349	α terpinyl acetate	0.66	±0.04	1284	isobornyl acetate	4.96	±0.03
1425	β caryophyllene	0.27	±0.02				
	Total Identified	99.05	±0.05		Total Identified	99.96	±0.01
	Not Identified	0.95	±0.05		Not Identified	0.04	±0.01
	Monoterpenes hydrocarbons	36.13	±1.37		Monoterpenes hydrocarbons	56.99	±0.04
	Oxygenated monoterpenes	60.99	±1.19		Oxygenated monoterpenes	38.02	±0.05
	Sesquiterpenes hydrocarbons	0.27	±0.02		Sesquiterpenes hydrocarbons	0.00	±0.00
	Oxygenated sesquiterpenes	0.50	±0.50		Oxygenated sesquiterpenes	0.00	±0.00
	Others	1.66	±0.10		Others	4.96	±0.03

Values are means (n = 3) and standard error.
